# Expert consensus on the diagnosis and treatment of heat stroke in China

**DOI:** 10.1186/s40779-019-0229-2

**Published:** 2020-01-13

**Authors:** Shu-Yuan Liu, Jing-Chun Song, Han-Ding Mao, Jin-Bao Zhao, Qing Song, Expert Group of Heat Stroke Prevention and Treatment of the People’s Liberation Army, and People’s Liberation Army Professional Committee of Critical Care Medicine, Da-Wei Liu, Kai-Jiang Yu, Jian-Guo Li, Hong-Yuan Lin, Sen Lin, Xi-Ming Dai

**Affiliations:** 1 Emergency Department, Sixth Medical Center, Chinese PLA General Hospital, Beijing, 100048 China; 2 Department of Critical Care Medicine, No. 908th Hospital of PLA, Nanchang, 360104 China; 3 Department of Critical Care Medicine, First Medical Center, Chinese PLA General Hospital, Beijing, 100853 China

**Keywords:** Heat stroke, Classic, Exertional, Diagnosis, Treatment, Expert consensus

## Abstract

Heat stroke (HS) is a fatal disease caused by thermal damage in the body, and it has a very high mortality rate. In 2015, the People’s Liberation Army Professional Committee of Critical Care Medicine published the first expert consensus on HS in China, *Expert consensus on standardized diagnosis and treatment for heat stroke*. With an increased understanding of HS and new issues that emerged during the HS treatment in China in recent years, the 2015 consensus no longer meet the requirements for HS prevention and treatment. It is necessary to update the consensus to include the latest research evidence and establish a new consensus that has broader coverage, is more practical and is more in line with China’s national conditions. This new expert consensus includes new concept of HS, recommendations for laboratory tests and auxiliary examinations, new understanding of diagnosis and differential diagnosis, On-site emergency treatment and In-hospital treatment, translocation of HS patients and prevention of HS.

## Background

Heat stroke (HS) is a fatal disease caused by thermal damage in the body, and it has a very high mortality rate. The incidence and risk of HS may far exceed estimations. Previous studies have shown that the main cause of sudden death in high intensity exercises is HS rather than cardiovascular events [1, 2] and that death caused by HS may exceed the total death caused by all natural disasters [3, 4]. In 2015, the People’s Liberation Army Professional Committee of Critical Care Medicine published the first expert consensus on HS in China, “Expert consensus on standardized diagnosis and treatment for heat stroke” [5], and the Expert Group of HS Prevention and Treatment of the People’s Liberation Army was established in 2016.

With an increased understanding of HS and new issues that emerged during the HS treatment in China, the expert consensus published in 2015 no longer meet the requirements for HS prevention and treatment. It is necessary to update the consensus to include the latest research evidence and establish a new consensus that has broader coverage, is more practical and is more in line with China’s national conditions. To this end, a new consensus work group and editorial committee for HS were established by the Expert Group on Heat Stroke Prevention and Treatment of the People’s Liberation Army and the Professional Committee for Critical Care Medicine of the People’s Liberation Army in September 2019. The meeting, held on October 20, 2018, generated a framework and the updated content for a new consensus. A preliminary draft of the new consensus was completed after compiling the opinions of each expert between December 15–20, 2018, and December 25–29, 2018, a seminar was held in Sanya with the participation of critical care medicine experts from China and members of the editorial committee, who carefully discussed the preliminary draft of the consensus and proposed revisions. The final version of the consensus was finalized on March 12, 2019, after 2 teleconferences.

This consensus is appropriate for the following population: clinical physicians from emergency medicine, critical medicine, neurology, hematology and other internal medicine departments at medical institutes (all care levels); military health personnel (basic level); medical emergency rescue personnel; and on-site medical support personnel at practices or competitions.

## Overview

When acting on the body, thermal damage can cause a series of pathophysiological changes, manifested by continuous mild to serious processes, including mild, moderate, and severe HS, collectively referred to as heat-induced disease. HS is the most severe type of heat-induced disease and has an extremely high mortality rate.

### HS

HS is an imbalance between heat production by and dissipation from the body caused by exposure to a hot environment and/or intense exercise, characterized by a core temperature of > 40 °C and abnormalities of the central nervous system, including changes in mental status, convulsions or coma and accompanied by life-threatening multiple organ damage. According to the differences in the cause of the disease and susceptible population, HS is classified into classic heat stroke (CHS) and exertional heat stroke (EHS).

### CHS

CHS is mainly caused by an imbalance between heat production by and dissipation from the body caused by passive exposure to a hot environment. CHS is commonly existed among young individuals, pregnant women and the elderly or individuals with chronic underlying disease or impaired immune function.

### EHS

EHS is mainly caused by an imbalance between heat production by and dissipation from the body caused by high intensity physical activity. EHS is common among healthy young people who exercise intensely during the summer, such as military officers and soldiers, athletes, firefighters and construction workers. Although EHS is more likely to occur in a hot and humid environment, sometimes environmental conditions are not necessary.

When describing heat-induced diseases in the relevant literature, concepts such as heat convulsion, heat syncope and heat exhaustion are commonly used [6–8]. Febrile convulsion refers to the occurrence of transient, intermittent muscle spasms during or after training that may be related to the loss of sodium salts. Heat syncope refers to the upright dizziness that occurs after standing or sudden change in posture for a long time in a hot environment, and it may be related to dehydration or poor self-regulation. Heat exhaustion is a clinical syndrome characterized by insufficient blood volume caused by loss of fluids during heat stress. These concepts are all specific pathophysiological presentations of thermal damage acting on the body or damage to specific organs or systems during the progression of heat-induced diseases, which can exist alone or coexist, and will not be emphasized in this consensus, because it provides little significance to distinguish these concepts.

## Epidemiology

According to the limited data, the incidence rate of CHS during summer heat waves is 17.6-26.5/100,000, the mortality of hospitalized patients is 14%-65%, and the mortality of ICU patients is > 60% [9–14]. EHS accounts for 8.6%-18.0% of patients with exertional heat-induced diseases, and the mortality rate is usually > 30% when combined with hypotension [13–16]. Notably, the incidence and mortality of HS reported at different times and locations are very different and may not be comparable. The possible reasons for these discrepancies include: 1) differences in environmental factors (such as the intensity and duration of a heat wave); 2) differences in labor intensity; 3) differences in diagnostic criteria; 4) whether on-site treatment in the early phase is appropriate. Additionally, CHS has a higher mortality rate, which may be related to existing underlying diseases.

China has a vast territory and the climate has been characterized by small regional temperature differences and prolonged presence of consistent temperature in the recent years. In some areas, the highest temperature in the summer can reach above 39 °C [17, 18]. Statistical data have shown that the incidences of HS and HS-induced deaths are increasing each year. However, the existing data are limited to some cities or provinces with small sample sizes, and as a result, they have limited reference value [19–22]. Currently, large-scale HS epidemiology data are still lacking in China.

The main risk factors for HS are a hot and humid climate and high intensity physical activity. CHS is mainly caused by hot and/or humid environmental factors and usually does not involve intense physical activity. Individuals with impaired thermoregulatory functions are more prone to CHS, including infants, the elderly, patients with chronic underlying diseases, individuals who require prolonged bed rest and obese individuals. The susceptibility factors for EHS are very different [23, 24]. It is not known why some individuals experience disease onset while others do not when exposed to the same environment. For many individuals, even if they have been exposed to an environment multiple times, EHS can still occur in that same environment with future exposure. Retrospective analysis of EHS cases in the Chinese military [25, 26] showed that dehydration, insomnia, psychological stress, underlying diseases, physical insufficiency, obesity, presence of acute inflammatory reactions (such as cold and diarrhea) and inadequate heat adaptation prior to training are susceptibility factors for EHS.

## Presentations of HS

### Presentations of different types of HS

#### CHS

The main source of heat is from the external environment (such as a heat wave). CHS is seen in elderly, young and weak individuals and patients with chronic diseases, and the onset is gradual. Prodromal symptoms of CHS are not easily identified, and the symptoms aggravate after 1-2 days with the presence of confusion, delirium and coma. The body temperature can reach as high as 40-42 °C, often accompanied by incontinence, heart failure and renal failure.

#### EHS

EHS is usually seen in healthy young people (such as military officers and soldiers, athletes, firefighters and construction workers), who, after a period of high intensity training or physically demanding labor, suddenly feel ill, such as extreme fatigue, persistent headache, uncoordinated movement, improper behavior, impaired judgment, flushing or paleness, nausea, vomiting and fainting, and may be accompanied by profuse sweating or no sweating. Subsequently, the body temperature rises rapidly above 40 °C, which causes severe central nervous system damage, resulting in delirium, epilepsy, decreased level of consciousness and coma [27]. Some patients do not have prodromal symptoms and suddenly faint or lose consciousness during exercise.

### Organ damage resulting from HS

#### Central nervous system

Functional impairment of the central nervous system is a main characteristic of HS, and severe damage can be present in the early stage of HS, manifested as delirium, drowsiness, epileptic seizure and coma. Other neurological abnormalities may also occur, including strange behavior, hallucinations, opisthotonus and decerebrate rigidity [28]. Some patients may have long-term damage to the central nervous system including lack of concentration, memory loss, cognitive impairment, language disorder and ataxia [29].

#### Coagulation

Direct heat damage and heat-related liver function abnormality can both cause impaired coagulation, which is clinically presented as skin petechiae, ecchymosis, puncture site bleeding, conjunctival hemorrhage, melena, bloody stool, hemoptysis, hematuria and intracranial bleeding. Patients complicated with disseminated intravascular coagulation (DIC) account for approximately 45% of patients with HS, which often indicates a poor prognosis [29]. When HS is complicated with coagulation impairment, thrombomodulin (TM), tissue plasminogen activator/plasminogen activator inhibitor-1 complex (t-PIC), thrombin-antithrombin complex (TAT) and plasmin-α_2_-antiplasmin complex (PIC) are elevated within hours of disease onset. Significant elevation of TM and t-PIC indicates vascular endothelial injury. TAT elevation indicates that procoagulation activity is initiated, and PIC elevation indicates hyperfibrinolysis. When HS is complicated with DIC, coagulation impairment can manifest as a progressive decline in platelet count (PLT) and fibrinogen (Fib), elevated or positive D-dimer and significant extension of prothrombin time (PT) and activated partial thromboplastin time (APTT). Abnormalities in these routine coagulation indexes (D-dimer, PLT, Fib, PT, APTT) often present 1–3 d after HS onset, and patients complicated with DIC may have significant abnormalities within hours.

Based on the theory of cell-based coagulation, thromboelastography (TEG) and coagulation and platelet functional analyzers can monitor the whole blood and detect early coagulation impairment. During HS, TEG can manifest as prolonged R and K time and reduced α angle and MA. Prolonged R time indicates reduced coagulation factor activity. Reduced α angle and prolonged K time indicate decreased fibrinogen function. Reduced MA indicates impairment of platelet function. LY30 > 8% indicates increased fibrinolytic activity, and CI < − 3 indicates a poor coagulation status. Coagulation and platelet function analyzers can show prolonged activated clotting time (ACT), decreased coagulation rate (CR) and reduced platelet function (PF). Prolong ACT indicates decreased coagulation factor function, decreased CR indicates decreased fibrinogen function, and reduced PF suggests platelet dysfunction.

#### Liver function

Severe liver damage is an important characteristic of EHS and is related to direct thermal damage, hypotension and visceral blood supply redistribution [30]. The most common clinical presentations are fatigue, anorexia and icteric sclera. Blood tests show rapid elevation of aspartate aminotransferase (AST), alanine aminotransferase (ALT) and lactate dehydrogenase (LDH), peaking at 3-4d (levels in some patients may peak at 2 weeks). AST can increase above 9000 U/L and ALT and LDH can increase above 10,000 U/L. These indexes gradually decrease with improvements in disease conditions. Increases in bilirubin levels relatively lag, usually starting 24-72 h after HS onset [5, 29]. Progressive jaundice mainly presented as elevation of indirect bilirubin often indicates a poor prognosis.

#### Renal function

HS patients often have renal damage, which is related to various factors including direct thermal injury, pre-renal damage caused by insufficient blood volume, renal hypoperfusion, rhabdomyolysis and DIC. Renal damage in HS patients is characterized by oliguria, anuria and dark urine (brown or soy sauce-colored urine). Acute oliguric renal failure occurs in 25%-35% of EHS patients and 5% of CHS patient [31].

#### Respiratory function

In the early stage of HS, the main presentations include shortness of breath and cyanosis of the lips. Approximately 60% of patients require mechanical ventilation, and 10% patients may develop acute respiratory distress syndrome (ARDS) [29].

#### Gastrointestinal function

In the acute phase of HS, high fever, reduced blood volume and gastrointestinal ischemia (diversion of gastrointestinal blood to the skin and muscle), oxidative stress and DIC may lead to gastrointestinal mucosal ischemia, intestinal wall edema, intestinal effusion and even bleeding [32, 33]. Gastrointestinal dysfunction can occur within 72 h of HS onset, presenting as nausea, vomiting, abdominal pain, diarrhea, and drainage-like stool, and patients with severe HS may have gastrointestinal hemorrhage, perforation and peritonitis [34, 35]. Intestinal endothelial damage can cause migrate in intestinal bacteria and toxins which may induce or aggravate the systemic inflammatory response to HS, intestinal infection or even shock, affecting the prognosis of HS patients [36, 37].

#### Cardiovascular function

Myocardial injury can occur on the first day of HS onset, and creatine kinase (CK), creatine kinase isoenzyme (CK-MB) and troponin I (cTnI) can increase to varying degrees. At HS onset, patients are in a highly dynamic status with an increased cardiac index (CI) and decreased peripheral vascular resistance (SVR). With cardiovascular damage aggravation, patients gradually transition to a low-dynamic status with decreased CI and elevated SVR. The main clinical presentations of cardiovascular insufficiency are tachycardia and hypotension; sinus bradycardia has also been reported in a few studies [38, 39].

#### Rhabdomyolysis

Rhabdomyolysis is a severe complication of HS that is related to abnormal mitochondria, abnormal glucose and lipid metabolism, and inflammatory myopathy. Rhabdomyolysis is characterized by muscle soreness, stiffness, muscle weakness, and dark urine, with muscle swelling and osteofascial compartment syndrome present in the late stage, eventually leading to acute renal failure [31]. Due to the involvement of thermal damage factors, HS-induced rhabdomyolysis is significantly different from general exertional rhabdomyolysis. The elevation of CK within 24 h of HS onset is usually not prominent but gradually increases and often peaks 5-7d after onset. The peak CK value is higher than that for exertional rhabdomyolysis and can reach as high as 400,000 U/L. Myoglobin (Mb) in the blood is often > 1000 ng/ml but can be 70,000-80,000 ng/ml or higher [5].

HS is normally characterized by nervous system impairment and complication resulting from multiple organ damage. The clinical presentations of patients may vary greatly and may even be atypical. For example, nervous system damage in some patients is not obvious in early stage, and significant symptoms may manifest after several days. In some cases, the initial body surface temperature is not significantly increased, but the core temperature is increased significantly. Additionally, the presentations of CHS and EHS are also different. EHS is often accompanied by severe rhabdomyolysis, early occurrence of acute renal injury, liver injury and DIC, which can appear within hours of onset and progress rapidly. Presentations of CHS may be confused with symptoms of underlying diseases, which may cause misdiagnosis. In addition, impaired consciousness of HS patients may be complicated with trauma or aspiration, making the clinical presentations more complicated.

## Laboratory tests

Patients with HS are critically ill, and laboratory tests should be conducted based on the general principles of critically ill patients to dynamically assess the disease conditions. Because HS is often accompanied by multiple organ dysfunction, laboratory tests should be as comprehensive as possible so that the changes in each indicator can be observed dynamically. If conditions permit, tests should be completed as soon as possible after onset and should include routine blood and urine, organ function assessment index, coagulation index, inflammation index, infection index, electrolytes, myocardial injury markers, rhabdomyolysis markers and arterial blood gas indicators, and blood culture should be carried out when necessary. Importantly, laboratory testing should not delay basic life support, nor should it delay rapid and effective reduction in body temperature.

Because HS has distinct characteristics different from general critical illnesses and because the disease progresses rapidly resulting in indicators at highly abnormal levels, it should be treated differently regarding the frequency of laboratory tests; higher frequency testing is especially necessary for coagulation function, liver function and muscle enzymes. Additionally, in the evaluation of coagulation function, more comprehensive tests can be performed using a coagulation and PF analyzer or thromboelastography (TEG), if available. Some indicators that are recommended for monitoring before the disease conditions are stable are provided in Table 1.

**Table 1:** Laboratory indicators and recommended frequency of monitoring in patients with heat stroke

Item	Indicators	Frequency
Blood routine	WBC, Neut%, PLT, Hb, HCT	1˗2×/d, increased frequency for patients with severe coagulation abnormalities or bleeding
Myocardial markers	cTnI, CK-MB, BNP	1˗2×/d
Rhabdomyolysis	CK, Mb	1×/d
Coagulation	PT, APTT, D-dimer, Fib, PT%	1×/4 h in the early stage until the indicators are stable
Renal function	Cr, Urea	1×/d
Liver function	AST, ALT, LDH, TBil	2×/d until the indicators are stable
Inflammation markers	CRP, PCT, IL-6	1×/d
Electrolytes	K^+^, Na^+^, Cl^−^, Ca^2+^	1˗2×/d and increased frequency according to the abnormalities
Blood glucose	BG	1×/2 h until the indicators are stale
Blood gas	pH, PO_2_, PCO_2_, HCO_3_^−^, BE, Lac	1×/4 h until the indicators are stale
Blood culture	Two aerobic and two anaerobic cultures	Patients with sustained high fever or suspected infection

## Auxiliary examinations

In the early stage of HS, there are usually not abnormal electrocardiography, chest radiography, ultrasound, computed tomography (CT) and magnetic resonance imaging (MRI) findings. However, with the progression of the disease, organ injuries are further aggravated, and changes related to tissue and organ injuries may occur. Reasonable auxiliary examinations are of great value for differential diagnosis, early detection of complications and dynamic assessment of disease conditions.

### Electrocardiography

Abnormal electrocardiography may continue for over 24 h in patients with HS, who often have abnormal sinus node dysfunction, tachyarrhythmia (sinus tachycardia, supraventricular tachycardia and atrial fibrillation), conduction abnormalities (right bundle branch block and intraventricular block), prolonged QT interval, and nonspecific changes in the ST segment. A small number of patients may manifest bradycardia [40, 41].

### Ultrasonography

Cardiac ultrasonography can provide information regarding ventricular morphology, contractility and diastolic performance, and heart chamber volume (such as measurement of the width and variability of the inferior vena cava), which can differentiate causes of myocardial damage [42]. Patients with severe HS may have a reduced cardiac ejection fraction accompanied by decreased ventricular wall motion. No abnormalities are usually detected in the early stage in abdominal ultrasonic examinations, and patients with severe liver damage may show thickened echoes of the liver parenchyma with uneven distribution.

### Cranial CT

For patients with HS with impaired consciousness, cranial CT can help detect and distinguish severe cerebral edema and hemorrhage. In the early stage of HS, there are usually no positive findings in cranial CT. Diffusive edema of the brain parenchyma may occur after 2-5 d. A previous study also showed, through cranial CT, brain edema and unclear boundaries between the gray matter and white matter at the early stage of HS [43]. Compared to brain edema caused by trauma and stroke, most cerebral edema in patients with HS is reversible and can gradually disappear after 7-10 d when the disease conditions stabilize [44]. Patients with impaired coagulation may have subarachnoid hemorrhage, intracranial hemorrhage or punctate hemorrhage, and infarcts may also occur.

### Cranial MRI

Patients with HS have a wide range of injured central nervous system sites; common sites include the cerebellum, basal ganglia, hypothalamus and the limbic system, and some less common sites are motor neurons in the anterior horn of the spinal cord, cerebral cortex and brain stem [43–46]. Different MRI sequences such as T_1_, T_2_, DWI and FLAIR can be used to determine various lesion types at different locations. MR image often shows abnormalities in the bilateral cerebella, caudate nucleus and subcortical white matter as well as uniform enhancement of the hippocampus. Patients with severe HS may have ischemic necrosis of the cerebellum or brain atrophy. MRI in the late stage of HS reveals ischemia and softening of the basal ganglia, globus pallidus, bilateral internal capsules, putamen and cerebellum. The cerebellum is an important target of heat damage in the central nervous system, and MRI from numerous HS patients indicate cerebellum atrophy. Long-term MRI follow up has revealed that residual damage to the central nervous system of survivors occurs mainly in the cerebellum and hippocampus. Proton magnetic resonance spectroscopy can detect cerebellar lesions that cannot be detected by conventional MRI [47].

### Electroencephalography

For HS patients with impaired consciousness, continuous EEG monitoring can help detect abnormal waves such as low-amplitude slow waves, epilepsy and longitudinal bipolar overlapping waves, but these EEG changes are usually nonspecific. The EEG changes that result from HS can often be completely recovered along with improvement in the disease status, which is significantly different from the abnormal EEG of primary neurological diseases [48–50]**.**

## Diagnosis and differential diagnosis

### Diagnosis

Currently, a unified standard for the diagnosis of HS is still lacking, and clinical diagnosis is largely based on medical history and clinical presentations. This consensus recommends the following diagnostic criteria based on relevant literature [9, 51–54] and the current treatment status in China:

Medical history information: 1) patients exposed to high temperature and high humidity environments; 2) patients subjected to high intensity exercise.

Clinical presentations: 1) functional impairment of the central nervous system (such as coma, convulsions, delirium and abnormal behavior);2) core temperature exceeds 40 °C;3) functional impairment of multiple organs (≥2) (such as liver, kidney, striated muscle and gastrointestinal tract);4) severe coagulopathy or DIC.

HS should be considered if the patient meets any of the medical history information together with any of the clinical presentations and if the symptoms cannot be explained by other reasons.

HS is a serious heat-induced disease, and extremely high body temperature is the first damaging factor. Most of the diagnostic criteria in the published literature used elevation of core temperature (usually rectal temperature) > 40 °C as a necessary condition for diagnosis. However, in practice, it is often impossible to measure rectal temperature at disease onset, or the highest rectal temperature is not detected (if the patients have been subjected to cooling treatment); the diagnosis of HS should not be delayed based on this. Additionally, core temperature cannot be replaced with surface temperature (usually axillary temperature). Because the accuracy of surface temperature is easily affected by many factors, it has limited reference value and cannot be used as a diagnostic condition. Tympanic temperature, rather than axillary temperature, is closer to the core temperature and can be used a reference.

The progression of patients from mild heat illness to HS is a continuous process of gradual aggravation. Intervention should be initiated when an abnormality is detected, and treatment should not be delayed for establishing an HS diagnosis. This consensus does not use core temperature as a necessary condition for the clinical diagnosis of HS to avoid a delay in necessary cooling treatments resulting from diagnostic issues. However, it does not mean that body temperature is not important. Body temperature is still one of the most important indicators of HS. If conditions allow, the patient’s body temperature, preferably the core temperature should be measured as soon as possible. Rectal temperature is recommended as the standard mode for acquiring core temperature. If the medical history and clinical presentations of a patient are consistent with HS, HS cannot be excluded simply because body temperature (including core temperature) does not exceed 40 °C.

### Differential diagnosis

Most patients with HS have altered consciousness accompanied with hyperthermia as the first symptom, which is complicated with corresponding symptoms related to multiple organ dysfunction. Clinically, HS should be differentiated from the following diseases.

#### Diseases of the central nervous system

1) Cerebrovascular disease: Cerebral hemorrhage, massive cerebral infarction and subarachnoid hemorrhage are common in patients with cerebrovascular disease, which may manifest as changes in consciousness, physical activity and verbal function. Patients with cerebrovascular disease often have underlying diseases including hypertension, diabetes and vascular malformations. In the early stage of cerebrovascular disease, there is generally not hyperthermia or organ damage outside the nervous system, and responsible lesions can be detected through imaging examinations. 2) Encephalitis and meningitis: According to different pathogens, encephalitis and meningitis can be bacterial, viral and tuberculous. The clinical symptoms are similar to that of HS, that is, characterized by high fever, headache and convulsions. However, the onset is not related to environmental factors and intense physical activity. It can be distinguished through a medical history. 3) Epilepsy: Epilepsy is a paroxysmal disease with a history of recurrent episodes and can occur during non-exercise time. Epilepsy is usually not associated with hyperthermia or multiple organ damage. Abnormal waves can be detected on electroencephalography (EEG).

#### Infectious diseases

HS can easily be misdiagnosed as shock and multiple organ damage caused by infectious diseases. However, infectious diseases often have corresponding presentations of infection, abnormal infection indicators and imaging changes, while HS can be differentiated by a specific history and susceptibility factors. The two diseases should be distinguished by a detailed medical history and physical examinations.

#### Metabolic diseases

Patients with hypoglycemic coma, hyperosmolar coma, hepatic encephalopathy or uremic encephalopathy may have impaired consciousness but generally no hyperthermia and no multiple organ damage in the short term. Symptoms of metabolic diseases can be alleviated by quickly correcting the primary disease.

#### Disturbances in water and electrolyte balance

Exertional hyponatremia, Which usually result from over consumption of fluids and inadequate intake of salt-free liquid after exercise, may cause hypotonic encephalopathy. The clinical presentations overlap with the nervous system presentations of HS. Electrolytes should be examined in a timely manner to distinguish the two diseases.

#### Malignant hyperthermia

Malignant hyperthermia is a subclinical hereditary muscle disease in which patients normally do not have abnormal presentations. Malignant hyperthermia is the occurrence of skeletal muscle tonic contraction, which generates large amounts of energy, resulting in a sustained and rapid increase in body temperature during general anesthesia when the patients are exposed to volatile inhalation anesthetics (such as halothane, enflurane and isoflurane) and depolarizing muscular relaxants (succinylcholine). In the absence of specific therapeutic drugs, it is difficult to control the increase in body temperature through general clinical cooling measures, which ultimately leads to death. Malignant hyperthermia is easily identified through a medical history.

## On-site emergency treatments

The keys to on-site treatment for HS patients are:1) rapid, effective and continuous cooling;2) rapid rehydration;3) effective control of restlessness and convulsions. Among these measures, the first point is the most important. In view of the critical condition and rapid progression, continuous cooling and evacuation should be implemented at the same time during the initial hours following heat stroke on-site treatment. If there is a conflict between continuous cooling and evacuation, on-site cooling should take precedence. Due to limited resources and extreme conditions on site, the following six critical treatment steps should be implemented to the best.

**(1)** Immediate removal from the heat. Whether affected by EHS or CHS, the patient should be quickly removed from the hot and humid environment (trainees should stop training immediately) and be transferred to a cool and ventilated location. The patient’s clothing should be removed as soon as possible to facilitate heat dissipation. If conditions allow, the patient should be transferred to an air-conditioned room with a room temperature of 16-20 °C.

**(2)** Quick measurement of body temperature. Fast and accurate measurement of body temperature is a prerequisite for effective cooling. Core temperature, instead of body surface temperature, should be measured on site because of the potential dissociation between the two in critically ill patients. Rectal temperature should be preferred; it can be measured by inserting a flexible rectal thermometer to a depth of at least 15 cm [55]. If core temperature (rectal temperature) cannot be measured on site, surface temperature (axillary or tympanic temperature) can be measured as a reference. HS cannot be excluded even if the axillary or tympanic temperature is below the diagnostic threshold; furthermore, body temperature should be measured every 10 min or monitored continuously.

**(3)** Active and effective cooling**.** Because the mortality rate is closely related to the degree and duration of hyperthermia [56], rapid, effective and continuous cooling is the primary treatment for HS patients. If initial cooling is delayed by 30 min, internal damage will continue even if the body temperature reaches the target. Previous studies have indicated that patients may not die if their core temperature is reduced to below 40.0 °C within 30 min [23, 57]. This consensus recommends that the core temperature should be reduced below 39.0 °C within 30 min and below 38.5 °C within 2 h. The cooling methods should be adapted to the local conditions and selected flexibly according to availability. Multiple methods can be used together. We recommend stopping the cooling measures or reducing the cooling intensity when the core temperature drops to 38.5 °C and maintaining a rectal temperature of 37.0-38.5 °C, in order to avoid hypothermia [6, 58]. The cooling measures should be restarted if the body temperature increases again. The following cooling methods, but not limited to those listed, can be used on site [59–69]. **1) Cooling by evaporation:** Effective cooling can be achieved by spraying the individual with cold water or misting the skin with water while applying air with a fan. A maximum cooling effect can be reached by maintaining the water temperature at 15-30 °C and the fan air at 45 °C, which prevents vasoconstriction by keeping the skin temperature at 30-33 °C. If conditions are limited, the patient’s skin can be covered as much as possible with gauze cloth (the patient should lie on his or her side to avoid aspiration), and room temperature water can be applied intermittently to the gauze so that the skin temperature is maintained at 30-33 °C, while fanning continuously. A moist towel or diluted alcohol can also be used to wipe the entire body while fanning continuously. In most circumstances, reducing temperature by evaporation is the easiest option suitable for both CHS and EHS patients and can be used as the primary option. **2) Immersion in cold water:** This method is mainly applied to EHS patients. Based on the principle of conduction cooling, the patient can be immersed, below the neck, in cold water (2-20 °C) using large containers (such as bath tubes, tarpaulins and sinks); this can be the most effective on-site cooling method. The cooling rate is 0.13-0.19 °C °C/min, and there is no significant difference in cooling effects of cold water at different temperatures. If cold water is not available, room temperature water (e.g., water at 26 °C) can be used for immersion. Special care should be taken to ensure that the patient’s head does not enter the water, and the respiratory tract should be protected to prevent aspiration and drowning. Adverse reactions of cooling by immersion in cold water include chills and restlessness, which usually occur after 9-10 min. In theory, chills and the accompanying skin vasoconstriction may reduce the effect of conduction cooling, but in reality, effective cooling can still be achieved. **3) Cooling by ice:** Using the principle of conduction cooling, the patient can wear an ice cap or use an ice pillow, and ice packs wrapped in gauze can be placed at places with abundant blood vessels and rapid heat dissipation, such as the neck, groin (the scrotum should be protected) and armpit. The ice packs should not be in place for more than 30 min each treatment. When applying ice, changes in local skin color should be monitored to avoid frostbite. Because this method leads to vasoconstriction in the skin, the skin should be massaged intensely while applying the ice. In fact, the effect of ice cooling is not ideal; the cooling rate is approximately 0.034 °C/min. **4) Cooling in vivo:** Body temperature can be reduced by stomach tube irrigation with normal saline at 4-10°C (rapid injection (total volume of 10 ml/kg within 1 min) through the gastric tube followed by aspiration after 1 min; the procedure can be repeated multiple times) or by rectal lavage (total volume of 200-500 ml injected at the rate of 15-20 ml/min at a depth of no less than 6 cm; it can be release after being placed for 1-2 min, and the procedure can be repeated multiple times.) The injection speed should not be too high during rectal lavage. Rapid intravenous injection of cold saline at 4 °C can also result in effective cooling, which is especially suitable for dehydrated EHS patients and is commonly used as a part of comprehensive treatment. Based on relevant literature, this consensus recommends infusion of normal saline at 4 °C at 25 ml/kg or a total volume of 1000-1500 ml within 60 min. The key to this method is to maintain a rapid infusion velocity; otherwise, the cooling effect cannot be reached. Core temperature should be monitored and should not decrease below 38.5 °C. If cold saline is not available on site, normal saline at room temperature can also be used for cooling. **5) Cooling by medication:** Due to thermoregulatory center dysfunction in the early stage of HS, the use of drugs, including nonsteroidal drugs and artificial hibernation compounds, to reduce body temperature during treatment on site is not recommended.

**(4) Rapid fluid recovery:** Venous access should be established quickly on site, preferably via thick peripheral veins. Establishment of a peripheral two-channel fluid path using a trocar instead of a steel needle is recommended because the latter is not easy to fix. A medullary ducts can also be established if available. Sodium-containing fluid (such as normal saline or Ringer’s solution) is preferred for infusion, which can replenish lost salt while rehydrating. This consensus recommends infusion of 30 ml/kg or a total volume of 1500-2000 ml within the first hour on site (if cooling by cold saline is initiated, the amount should be included in the total volume calculation). The infusion rate should be adjusted according to the patient response (such as blood pressure, pulse and urine volume) after the first hour so that the urine volume of patients without renal failure is maintained at 100-200 ml/h while avoiding fluid overload. Infusion of a large amount of glucose should be avoided to prevent a rapid reduction in blood sodium in a short period of time that may aggravate nerve damage [70].

**(5) Airway protection and oxygen therapy:** The head of a patient in a coma should be turned to one side to keep the airway open. Airway secretions should be cleared in a timely manner to prevent vomiting and aspiration. Water should not be provided to unconscious patients. If vomiting has occurred, the oral secretions should be cleaned as soon as possible. Tracheal intubation should be performed as soon as possible for HS patients who require airway protectio n[71, 72]. If intubation is not available on site, the airway should be kept open/be continuously opened by hand, orapharyngeal/nasopharynx airway, and then, call for support as soon as possible. Pulse oximetry (SpO_2_) should be monitored continuously during on-site treatment if conditions permit. Nasal cannula oxygenation should be used to maintain SpO_2_ ≥ 90% [51]. If the nasal cannula fails to meet the oxygen level, a mask should be used for oxygenation.

**(6) Control of convulsions:** Convulsions and restlessness not only interfere with cooling treatments but also increase heat production and oxygen consumption, which aggravates damage to the nervous system. It is extremely important to control convulsions and restlessness on site. Sedative drugs can be given to keep the patient in a sedated state and prevent accidental injuries, such as tongue biting. Restless patients can be injected with 10-20 mg of diazepam intravenously within 2-3 min. If intravenous injection is difficult, diazepam can be injected intramuscularly. If the convulsions are not ameliorated after the first dosage, 10 mg of diazepam can be injected intravenously after 20 min; total dose within 24 h should not exceed 40-50 mg. If convulsions are not controlled properly, intramuscular injection of 5-8 mg/kg phenobarbital can be used in addition to diazepam.

## Translocation

Patients with confirmed or suspected HS should be transported as soon as possible after on-site treatment to the nearest hospital that has HS treatment experience in order to receive advanced treatments. Rapid, effective and continuous cooling is the primary treatment of HS. Even if a patient with HS is evacuated, body temperature should be reduced effectively and continuously during transportation.

### Evaluation prior to translocation

Benefits and risks should be assessed prior to evaluation of HS patients, and translocation is only appropriate when the benefits outweigh the risks. Prior to the translocation, the patient’s condition, including consciousness, heart rate, blood pressure, oxygen saturation, presence or absence of airway obstruction and arrhythmia, should be assessed to determine whether the patient is suitable for translocation. If not, the patient’s conditions should be corrected before translocation. This consensus recommends the following indications for translocation: 1) body temperature > 40 °C;2) body temperature > 40 °C 30 min after on-site treatment to reduce the temperature (e.g., moving the patient to a cool location, spraying the patient with water, patient immersion and fanning);3) no improvement in consciousness;4) the site lacks necessary conditions for treatment.

### Management of the translocation

For critically ill patients with HS, the fastest achievable means of transportation should be used for safe and efficient translocation, including air, water and land vehicles. Currently, ambulances are still the most conventional vehicles for patient translocation. Medical personnel with experience in the treatment of critically ill patients, including at least 1 physician and 1 nurse, should accompany the patient during translocation. First aid equipment and medicines should be checked prior to translocation. The following should be done during translocation: 1) The body temperature of the patient should be monitored closely every 0.5-1.0 h. Rectal temperature should be assessed if conditions allow, and vital signs should be monitored and recorded. 2) The body temperature of the patient should be lowered continuously and effectively, which should not be delayed because of translocation. The following measures can be taken according to the available conditions: adjust the air conditioning temperature in the ambulance to the coldest setting or open the windows, reduce body temperature by wiping the entire body with cold water and continuous fanning, reduce body temperature by putting ice on the body and treat conscious patients with 4-10 °C normal saline by infusion or oral administration.

## In-hospital treatment

### Targeted temperature management (TTM)

TTM refers to a therapeutic strategy that aims to achieve and maintain a specific core temperature in particular patients to improve clinical outcomes [73]. In recent years, people have gradually realized the importance of accurate body temperature management in the treatment of critically ill patients [74], which may be especially important for patients with HS, and TTM should be implemented for patients throughout hospitalization. The core temperature of hospitalized patients should be measured immediately. For patients who have been treated on site or during translocation to reduce body temperature, treatment should be continued if the core temperature is still higher than the target temperature. If the core temperature has reached the target temperature at the time of admission, body temperature should be monitored continuously so that it is not too low or does not increase again.

#### Continuous monitoring of body temperature

Continuous and accurate monitoring of body temperature is the basis of TTM. Surface temperature measurements (ear canal, mouth, ear drum, armpit and tempus) are not recommended to estimate the core temperature of hospitalized patients because these methods cannot accurately reflect core temperature and may be misleading. Esophageal temperature can best represent cardiac and brain blood temperature and responds rapidly to acute temperature changes; therefore, it can be used to estimate core temperature. However, the method for obtaining esophageal temperature is complicated. Rectal temperature is less invasive and easier to measure and can reflect the temperature of important organs in the abdomen. This consensus recommends that rectal temperature be used to monitor the core temperature of HS patients. If a flexible thermometer is used, to obtain more accurate core temperature, the thermometer should be inserted to a depth of 15 cm through the anus. Core temperature should be monitored continuously or measured at least once every 10 min for HS patients before the disease condition is stabilized. When measuring core temperature, the damage to the rectum and the adjacent tissue should be avoided [55, 58].

#### Effective body temperature control

According to different heat dissipation principles, hospitalized patients have access to more cooling measures, which can be used alone or in combination depending on hospital conditions, to achieve more accurate body temperature control. Cooling measures used for on-site emergency treatment can all be used in a hospital setting (refer to on-site emergency treatment). The following are other cooling options [51, 75, 76]: 1) Temperature-control blankets are convenient to use, simple and effective. The patient lies flat on the cooling blanket and the starting temperature of the blanket is set to 38.5 °C, the shutdown temperature is 37.5 °C, and the surface temperature of the blanket is 4 °C; An ice cap or ice pillow can be used simultaneously to achieve rapid cooling. 2) Cooling can be conducted by intravascular heat exchange, including extracorporeal machines, pumps and intravenous catheters. Intravascular cooling method is rapid and accurate and has been successfully used for the treatment of HS. 3) Cooling can be achieved by drugs. Commonly used nonsteroidal antipyretic and analgesic drugs (such as aspirin and indomethacin) in clinical practice are not appropriate for rapid cooling in the early stage of HS and can increase risk of liver toxicity. Therefore, these drugs are not recommended. Dantrolene can inhibit excess calcium release from the endoplasmic reticulum and is the only effective medicine for the treatment of malignant hyperthermia caused by persistent muscle spasm. However, persistent muscle spasm is uncommon among patients with HS, and as a result, the use of dantrolene for patients with HS is still controversial. 4) Another method is continuous blood purification (CBP). Blood purification is an important measure for organ support in HS patients and can also achieve accurate intravascular cooling. Because the blood is taken out of the body and dialysis/replacement fluid is used, rapid cooling can be achieved by controlling the temperature of the external warming device and simultaneously controlling the temperature of the ward. Cooling is faster with a faster blood flow rate and a larger amount of the replacement fluid. Continuous veno-venous hemodiafiltration (CVVHDF) mode enables a higher flow rate of the dialysis/replacement fluid and faster cooling. In the late stage, HS is often complicated with infection, and thus, the fever mechanism is not consistent with that of early stage HS. Therefore, different cooling strategies should be selected.

#### Maintenance of target temperature

There is currently no evidence that establishes the optimal target temperature for cooling treatment. Conventionally, core temperature may be reduced further after discontinuation of cooling measures [58]. To prevent potential risks induced by hypothermia, such as arrhythmia and coagulopathy, the target temperature of cooling should be set slightly higher than normal body temperature [59]. Most studies recommend termination of cooling by cold water immersion at 38.6-39.0 °C and by evaporation at 38 °C [6, 8, 59, 77]. This consensus recommends maintaining a rectal temperature at 37.0-38.5 °C as the core temperature goal.

#### Induced subhypothermia

Subhypothermia therapy has shown potential for the treatment of some neurological and cardiovascular emergencies, including acute stroke and cardiac arres t[78]. However, the efficacy of this therapeutic strategy has not been confirmed for the treatment of CHS or EHS and is mainly reported in empirical cases [79–81]. Due to limited evidence and experience, there is currently no recommendation for the use of subhypothermia therapy in HS.

### Airway management and respiratory support

When actively controlling core temperature, the patient’s airway should be kept open. Most HS patients have impaired consciousness and require airway protection. Tracheal intubation should be actively performed early. This consensus recommends using the following signs for determining the need for tracheal intubation: 1) impaired consciousness and symptoms of deliriums, irritability, muscle tremors and convulsions; 2) deep sedation; 3) excessive airway secretion with impaired drainage; 4) risk or occurrence of aspiration; 5) respiratory failure and progressive deterioration of the oxygenation status; 6) instable hemodynamics and poor response to fluids and vasoactive drugs. The decision to proceed with endotracheal intubation and mechanical ventilation should be based mainly on the assessment of the clinical conditions rather than arterial blood gas results because it is difficult to define normal arterial blood gas values in the presence of high fever. Additionally, early tracheotomy is not recommended because HS is usually complicated with severe coagulopathy and aggressive tracheotomy may lead to uncontrolled local bleeding.

There is no definitive conclusion on the target of respiratory support for HS patients. This consensus recommends that an SpO_2_ target value of 90%-99% or arterial oxygen partial pressure (PaO_2_) target value of 60-100 mmHg. Excessive oxygen partial pressure may also be harmful. PaCO_2_ should be maintained between 35-45 cmH_2_O or at baseline values [51]. Oxygen therapy should be selected according to the patient’s condition. Unintubated patients can choose nasal catheter oxygen or mask oxygen. If the condition of the patient does not improve with mask oxygen, nasal high-flow humidification oxygen therapy or noninvasive positive pressure ventilation can be used. Patients should meet the following requirements for the use of noninvasive positive pressure ventilation: 1) awake and cooperative; 2) stable hemodynamics; 3) do not require endotracheal intubation protection (no aspiration, severe gastrointestinal bleeding, excessive airway secretions and poor drainage); 4) no facial trauma affecting the use of a nasal/facial mask; 5) can tolerate nasal/facial mask.

Most HS patients need to undergo endotracheal intubation as early as possible [71, 72], and invasive mechanical ventilation is the first choice for respiratory support for most patients. Despite a lack of consensus standards for indications and parameters for mechanical ventilation in HS patients, most scholars believe that mechanical ventilation should be performed as soon as possible. This consensus recommends the use of a protective ventilation strategy for mechanical ventilation, including limiting the tidal volume to ensure a platform pressure ≤ 30 cmH_2_O and setting the PEEP to an appropriate level.

### Circulation monitoring and management

Circulation impairment in HS patients is mainly characterized by hypovolemia and cardiac dysfunction through the following mechanisms [82–85]. 1) When HS patients suffer heat stress blood flow is distributed to the peripheral circulation to accelerate heat dissipation, leading to an insufficient effective circulating blood volume. 2) Excessive sweating leads to a substantial loss of fluid, causing a compensatory increase in heart rate. 3) High fever causes direct myocardial injury. 4) Secondary systemic inflammatory reactions further lead to myocardial damage. Blood pressure, heart rate, respiratory rate, SpO_2_, central venous pressure (CVP), blood gas (including central venous blood gas S_cv_O_2_ and P_v-a_CO_2_), lactic acid, urine volume per hour and urine color should be monitored continuously in hospitalized HS patients. For the precise hemodynamic management, patients can undergo invasive monitoring such as invasive arterial pressure or pulse-induced contour cardiac output (PiCCO) if available.

On the basis of on-site fluid resuscitation, hospitalized HS patients can further be evaluated for the circulatory status and tissue perfusion. In the presence of circulatory instability or tissue hypoperfusion, cardiac function (bedside ultrasound is recommended) and fluid reactivity (fluid challenging test or passive leg lift test) should be evaluated. Whether to continue fluid resuscitation is determined according to the fluid reactivity results. During resuscitation, blood pressure, heart rate, CVP, S_cv_O_2_, P_v-a_CO_2_, urine volume, and lactic acid level should be monitored dynamically and tissue hypoperfusion should be examined dynamically to determine improvements. While adequate fluid should be resuscitated, fluid overload should also be avoided.

There is currently no evidence from large-scale clinical studies for the use of vasoactive drugs in patients with HS. Considering the sepsis-like inflammatory response in the pathogenesis of HS, HS-induced shock has characteristics of distributed shock. This consensus recommends referring to the drug use strategy for septic shock [86]. If tissue hypoperfusion is still present in patients after sufficient fluid resuscitation, vasoactive drugs should be used as soon as possible. Norepinephrine is preferred, and norepinephrine combined with epinephrine can be used if the standards are not met. Dopamine can be used as an alternative for patients with low risk of tachyarrhythmia or bradycardia. For patients who require a vasopressor, an MAP of 65 mmHg is recommended as the initial target for resuscitation. If hemodynamics is still not stable for sufficient fluid resuscitation and vasoactive drug treatment, intravenous hydrocortisone is recommended at a dose of 200 mg/d.

### Treatment of coagulopathy

#### Substitution therapy

Goal-directed replacement therapy is recommended for the treatment of coagulopathy in patients with HS; this therapy is guided by monitoring regular coagulation indicators or using whole blood monitoring devices such as TEG and Sonoclot analyzers [54, 87]. Replacement therapies include the following:

**(1) Supplementation of coagulation factors:** Fresh frozen plasma should be infused intravenously at 15-30 ml/kg as soon as possible if the PT or APTT is extended by > 1.5× or TEG R is > 10 min or the ACT based on coagulation and PF analysis > 180 s (natural whole blood). Coagulation indicators can be monitored dynamically after infusion to determine the amount of additional infusion needed. If an excessive fluid load is present in the patient, coagulation factors can be supplemented in combination with prothrombin complex concentrate [88]. Cryoprecipitate (10 ml/kg) or human fibrinogen concentrate (Fib, 30-50 mg/kg) can be infused if Fib level < 1.5 g/L or the TEG functional Fib indicator FF_MA_ is < 10 mm or the CR based on Sonoclot analysis is < 10. Coagulation indicators can be monitored dynamically after infusion to determine the amount of additional infusion needed, and plasma Fib level should be maintained at least at 1.5 g/L [89].

**(2) Supplementation of platelets:** One therapeutic unit of machine-collected platelets can be infused if the platelet count is less than 50 × 10^9^/L or the TEG MA is < 50 mm and FF_MA_ is > 10 mm or the PF based on Sonoclot analysis is < 1. In theory, one unit of platelets can increase the platelet count by (10-20) × 10^9^/L; platelet-related indicators can be monitored dynamically one hour after infusion to determine the amount of additional infusion needed [90].

**(3) Supplementation of recombinant coagulation factor VII:** If hemorrhage is not effectively stopped by active replacement therapy and a hypocoagulant state is indicated by conventional coagulation monitoring or whole blood monitoring, recombinant coagulation factor VII (rVII) can be used. The following conditions should be met when using rVII to stop bleeding: 1) acidosis, hypothermia and hypocalcemia have been corrected; 2) hematocrit > 24%, platelet count > 50 × 10^9^/L and Fib level > 1.5 g/L. The initial dose of rVII should be 200 μg/kg, and depending on the presence of bleeding, 100 μg/kg rVII can be administered at an interval of 2 h. The drug can be discontinued according to the presence of bleeding and the results of blood coagulation tests [91].

#### Anticoagulant therapy

##### Timing of anticoagulation

The aim of anticoagulant therapy is to reduce the excessive consumption of blood coagulation substances by anticoagulation to interrupt the development of DIC. However, evidence-based data are still lacking for the use of anticoagulant therapy in patients with HS. This consensus recommends the use of coagulation molecular markers combined with TEG or other whole blood function monitoring to determine the appropriate timing for anticoagulation. For example, anticoagulation therapy can be initiated simultaneously with target-directed replacement therapy when the thrombin-antithrombin complex (TAT), D-dimer (DD), fibrin degradation products (FDPs) and plasmin-α_2_ anti-plasmin complex (PIC) are significantly elevated and whole blood function monitoring results show significant hypocoagulability combined with significant organ dysfunction. Coagulation function should be monitored during anticoagulation treatment to assess anticoagulation efficacy and the risk of bleeding. If there is active bleeding (such as intracranial hemorrhage and gastrointestinal bleeding), evaluation of the appropriate timing for anticoagulant therapy should be conducted after controlling the bleeding [92].

##### Selection and dose of anticoagulant drugs

Parenteral anticoagulant drugs should be selected and preferably administered intravenously. The following drugs can be used [93–95]. **1) Unfractionated heparin (UFH):** UFH is recommended as a first-line treatment because it has the advantages of a short half-life, easy monitoring and neutralization by protamine. UFH should be administered intravenously and according to the coagulation and organ function status. A maintenance dose of 1-8 (U/kg·h) should be used, and the dosage should be adjusted according to the APTT or TEG R time or the change in ACT value of Sonoclot analysis over the baseline value. Heparin dosage can also be selected at an R/Rh ratio of 1.5× according to TEG heparinase comparison analysis. Excessive heparin may be antagonized by protamine. UFH may cause heparin-induced thrombocytopenia (HIT). After administration of UFH, if the platelet count decreases more than 50% of the baseline value and/or if there are signs of arteriovenous thrombosis, HIT can be excluded or detected by the 4 T score or HIT antibody. UFH should be discontinued in patients with highly suspected or confirmed HIT, and non-heparin anticoagulant drugs should be used. **2) Low molecular weight heparin:** Due to the long half-life, it is difficult to adjust and monitor the dose of low molecular weight heparin, which is suggested as an alternative to heparin for continued anticoagulation therapy after alleviation of acute coagulation function impairment. An initial dose of 1 mg/kg q12 h (intravenous or subcutaneous injection) is generally recommended and the dose should be adjusted by anti-Xa activity. The target range is 0.6-1.0 U/ml. Because low molecular weight heparin is metabolized through the kidneys, patients with renal dysfunction should be monitored closely. **3) Argatroban:** Argatroban can be used if patients are allergic to heparin or if HIT occurs. Argatroban is a direct thrombin inhibitor that is metabolized in the liver and can cause significant thrombin time (TT) prolongation. The recommended initial dose of argatroban is 0.2-0.55 μg/(kg∙min) through intravenous infusion, and its dose can be adjusted dynamically according to the APTT or TEG R time or the change in ACT value over the baseline value. **4) Bivalirudin**: Bivalirudin is a direct thrombin inhibitor; its effective anticoagulant component is a hirudin derivative fragment. It exerts an anticoagulant effect by directly and specifically inhibiting thrombin activity. Bivalirudin has a short-acting effect (half-life 25-30 min), is reversible, and can be used in patients with HIT or suspected HIT. The initial dose is 0.05 (mg/kg·h), and the dose can be dynamically adjusted according to the APTT or TEG R time or the change in ACT value over the baseline value.**5) rAT and rTM:** Currently, rAT and rTM are recommended in the Japanese guidelines for the treatment of DIC. These drugs are not available in China.

##### Timing of drug discontinuation

Anticoagulation drugs can be discontinued when coagulation function is corrected, that is, PLT is maintained, without intervention, at a normal level, and coagulation indices, such as TAT, DD, FDP and PIC, and whole blood monitoring results are normal. Changes in coagulation function should be monitored after drug discontinuation. If coagulation function becomes impaired again, the cause and indications for drug treatment should be assessed. Anticoagulant therapy should be continued for at least 3 months if the patient’s condition is complicated with deep venous thrombosis. Intragastrointestinal anticoagulant drugs such as warfarin, rivaroxaban or dabigatran may be considered.

### Central nervous system injury and brain protection

Injury to the nervous system is especially prominent among the organ injuries of HS patients. The brain is one of the most easily affected organs in the early stage of HS, and it recovers slowly. The incidence rate of permanent nerve injury among surviving patients is 20%-30% [96]. The detailed mechanism of central nervous system injury is still not clear, and currently, it is believed to be related with the following mechanisms [46, 97–100]: 1) direct damage by high heat - The central nervous system is sensitive to heat toxicity, with the most vulnerable parts being the cerebellum and cerebral cortex; 2) secondary hypoxia - convulsions, aspiration and asphyxia in HS patients are likely to lead to hypoxia of the brain and are potential factors that aggravate brain damage; 3) ischemic necrosis -microthrombus formation caused by elevated intracranial pressure and decreased cerebral blood flow can lead to extensive ischemic brain damage; 4) secondary bleeding - severe coagulopathy or inappropriate anticoagulant therapy can cause cerebral hemorrhage.

Currently, specific methods for treating brain damage in HS patients are not available. The key to clinical treatment is to address the above factors that cause brain damage, prevent or alleviate further damage and promote recovery. The following are the main treatment strategies [51, 101–105]. **1) Rapid and effective cooling** is the most important measure for alleviating injury to the central nervous system, and a faster cooling rate is more beneficial for the prognosis of HS patients. **2) Protection of the airway by endotracheal intubation** is required for patients who are in a persistent coma or have aspiration, suffocation, or reduced cough reflexes. These patients should be provided with respiratory and circulatory support to prevent cerebral hypoxia. **3) Effective analgesia and sedation:** If HS patients are restless and have convulsions, sedatives that are fast acting and have strong efficacy and few side effects can be used, such as propofol and benzodiazepines. For patients with uncontrollable convulsions, neuromuscular relaxants can be used in combination during the early stage. Dexmedetomidine may be considered for patients with delirium. In addition to its sedative, analgesic and anti-anxiety effects, dexmedetomidine has myocardial protection and brain protection effects, making it especially appropriate for HS patients. **4) Dehydration by mannitol** is beneficial for brain protection. Mannitol can be used at 125 ml, 2-3 times/d for 7-10 days assuming there is adequate cerebral perfusion. Dehydration treatment should be initiated as soon as possible after stabilization of circulation in HS patients; however, there is currently no sufficient evidence to support it. **5) Hyperbaric oxygen therapy** can effectively improve the inflammatory response, increase blood flow in brain tissue and reduce oxidative damage. Hyperbaric oxygen therapy can improve nerve function especially for patients with residual impairment of the central nervous system in the late stage. **6) Other treatments.** It is not clear whether other treatments such as hyperventilation, CRRT, hormone therapy, neurotrophic nutrition and routine intracranial pressure monitoring are beneficial for neurological outcomes, and recommendations are currently not available.

### Treatment of liver damage

Liver damage is a common complication of HS and is also one of the important causes of death of HS patients [106]. The liver is the sentinel organ of heat shock, and evidence of liver damage can be detected within hours of HS onset. After the occurrence of HS, blood flow in the liver is reduced, and due to concurrent DIC, an extensive microthrombus is formed in the liver, leading to ischemia and hypoxia [107]. Fulminant hepatic failure can occur in very few cases, with an incidence rate of approximately 5%, and hypophosphatemia can be used as a predictive biomarker [108, 109]. The risk of hepatic failure may be significantly increased if antipyretics (such as paracetamol) are used in the early stage to treat HS [30, 108]. The pathological characteristics of liver damage in patients with HS is degeneration and necrosis of the hepatic lobules accompanied by substantial damage, but usually, no significant abnormality is detected in abdominal ultrasound and liver CT in the early stage of HS. The extent and changes in liver damage can be dynamically reflected by liver function tests. AST, ALT and LDH are elevated rapidly within hours of disease onset and reach a peak at 3-4d. Bilirubin usually begins to rise at 24-72 h after onset. If there is impairment of phosphoenolpyruvate carboxykinase, the patients may have hyperglycemia or hypoglycemia. Liver damage can also lead to reduced synthesis of coagulation factors and aggravates coagulopathy.

Currently, no evidence has confirmed for specific liver-protecting drugs. Rapid cooling in the early stage and supportive treatment are still the most effective measures. Mild patients should rest in bed, inhale oxygen, maintain a stable internal environment and be closely monitored for changes in liver function indexes. The following drugs can be considered: glutathione, diammonium glycyrrhizinate, polyene phosphatidylcholine and ademetionine. For critically ill patients with rapidly elevated bilirubin and DIC, artificial liver therapy should be performed as early as possible [110], such as plasma exchange or plasma diafiltration (PDF); however, the evidence is not directly from HS patients. If the disease progresses rapidly, mixed blood purification treatments with alternate use of plasma replacement or PDR and CRRT (CVVHDF is recommended) are recommended, but the duration depends on the patient’s condition. Liver transplantation can be considered for some HS patients with liver failure. However, current data are limited to certain EHS cases with limited effect, and therefore, recommendations cannot be given [111].

### Protection and treatment of gastrointestinal function

Damage to gastrointestinal function is common among HS patients. Gastrointestinal symptoms (such as nauseas, vomiting and diarrhea) are early presentations of HS, especially EHS. Early effective cooling and active fluid resuscitation are the most important measures for the alleviation or prevention of gastrointestinal damage. Clinically, the main measure for gastrointestinal function protection is early enteral nutrition.

Enteral nutrition and probiotics supplementation are not recommended when hemodynamics are not stable and at the early stage of the disease (within 72 h). After 72 h, enteral nutrition should be provided as soon as possible if the patient’s hemodynamics and internal environment are stable and gastrointestinal bleeding and paralytic bowel obstruction are absent. Low-dose enteral nutrition can be considered when hemodynamics is stable, the presence of intestinal ischemia should still be cautiously assessed. Low-dose enteral nutrition (unrelated to the extent of hepatic encephalopathy) can be used for patients with HS complicated with severe hepatic dysfunction and hyperbilirubinemia after controlling acute metabolic disorder (with/without liver support) [112].

Food can be provided orally to conscious patients who can first drink a small amount of warm water, gradually transitioning to a liquid diet, semiliquid diet and eventually normal diet if coughing and abdominal distension are absent. Tube feeding routes (nasal tube or nasal jejunal tube) can be chosen for patients who cannot be fed orally. During tube feeding, the head of the bed should be elevated to 30-45° to reduce the risk of aspiration pneumonia. The temperature of the nutritional agent should be maintained at 37-40 °C, and the principles of “from less to more”, “from slow to fast” and “from diluted to concentrated” should be followed during infusion. Continuous infusion using a nasal feeding pump generally starts at 20 ml/h and can be gradually increased if tolerated by the patient. For patients who are intolerant, the infusion speed can be reduced to a tolerable level and then increased gradually.

Different enteral nutrition agents can be selected according to the extent of liver and renal functional damage of the patients and can be divided into short peptide agents and whole protein. Patients with gastrointestinal dysfunction should gradually transition from short peptide agents to whole protein agents. Low calorie feeding at 20-25 kcal/(kg·d) should be used for critically ill patients. Other nutritional agents (such as glutamine, probiotics or prebiotics) only show some benefits in some critically ill patients [112, 113]. There are currently no recommendations regarding routine supplementation with nutritional agents for HS patients. Because the majority of HS patients have severe liver and renal impairment, glutamine should be used with caution.

### Treatment of rhabdomyolysis (RM)

RM is a common complication of HS and is more common among patients with EHS. Intense exercise or high fever may lead to striated muscle hypoxia and impaired cell metabolism and cause necrosis and rupture of muscle fibers. As a result, the contents in muscle cells (such as myoglobin, CK and small molecules) are released into the extracellular fluid and blood, leading to a clinical syndrome characterized by “myalgia, weakness and dark urine”. Large amounts of myoglobin can occlude the renal tubules in an acidic environment, causing damage to renal function and accelerating the development and progression of multiple organ dysfunction syndrome (MODS) [114].

Currently, there is lack of uniform diagnostic criteria for RM caused by HS. This consensus suggests that RM can be clinically diagnosed in the presence of any of the following in HS patients. 1) Significant elevation of CK that exceeds normal peak values by 5 fold of more or is more than 1000 U/L. CK may be the most sensitive indicator for the diagnosis of RM at present,[115] and elevated level is related to acute renal injury. A previous study showed that the changes in CK values at 48 h after hospital admission are effective indicators for determining the prognosis of HS patients [116]. 2) The levels of blood and urinary myoglobin are elevated significantly. Myoglobin is a specific but insensitive marker because its half-life is only 1-3 h and it is easily cleared. Additionally, urine turns red if the concentration of urinary myoglobin exceeds 150 ng/ml. However, existing urine test paper methods cannot distinguish between myoglobin and hemoglobin; therefore, the use of urine test paper methods for the diagnosis of RM or myoglobinuria is not recommended. 3) Patients have occult blood in their urine, but red blood cells are not detected under the microscope.

The main cause of HS-induced RM is high fever and/or excessive muscle exercise. Therefore, actively reducing the core temperature of patients and controlling muscle twitching are key to preventing continuous muscle injury. On this basis, different therapeutic strategies can be chosen according to the renal function and urine volume of the patient.

1) Fluid therapy and alkalizing urine: Fluid supplementation is the basis for the treatment of RM to prevent secondary renal injury. Fluid therapy should be started immediately once RM is diagnosed or even suspected. Initially, normal saline or 0.45% saline (5% glucose solution mixed with normal saline at a 1,1 ratio) can be used, and the initial infusion is usually more than 500 ml/h to maintain the urine volume at 200–300 ml/L in patients without renal injury. A 5% NaHCO_3_ solution should be infused to maintain urine pH above 6.5, but arterial blood gas pH should not exceed 7.5 [117]. The above fluids should be included in the management of total body fluids of HS patients. Hemodynamics and urine volume should be closely monitored during fluid supplementation to prevent fluid overload.

2) Diuretic application: Currently, there is still controversy regarding the application of the osmotic diuretic mannitol and loop diuretic furosemide. Although animal experiments investigating RM have shown that mannitol can reduce renal injury by reducing renal oxidative stress and recover urine volume, a retrospective clinical study did not find an effect of mannitol on renal function [118]. This consensus does not provide any recommendations on the use of diuretics but emphasizes that diuretics should not be used in patients who have not achieved sufficient fluid resuscitation.

3) CBP: Blood purification treatment is recommended for patients with refractory hypercalcemia, severe hyperkalemia, metabolic acidosis and anuria. The molecular weight of myoglobin is 17.6kD, and the myoglobin in blood can be effectively removed using a high-retention filter/dialyzer with a pore size of 20 nm [119]. Continuous veno-venous hemofiltration (CVVH) or continuous veno-venous hemodiafiltration (CVVHDF) can be used for treatment. Continuous veno-venous hemodialysis (CVVHD) should be avoided because it cannot effectively clear myoglobin. For patients with adequate urine output, preventive blood purification or blood purification for simply removing myoglobin is not recommended [120].

### CBP

CBP purifies blood and treats disease by removing the patient’s blood and passing it through a blood purification device to remove pathogenic substances. Methods of blood purification include hemodialysis, hemofiltration, blood perfusion, plasma exchange and immunoadsorption.

Blood purification is of great significance in the treatment of HS patients [121–123]: 1) It can achieve effective intravascular cooling and is one of the most effective means of cooling in hospitalized HS patients. 2) It can help achieve precise volume management for patients with HS complicated with acute renal injury. 3) It corrects electrolyte imbalance and acidosis and maintains a stable internal environment. 4) It clears pathogenic media (such as CRRT for the treatment of rhabdomyolysis and plasma exchange for the treatment of hyperbilirubinemia and alleviates secondary injury.

This consensus suggests that for HS patients, the timing of initiating blood purification treatment is more important than for other critically ill patients. CBP can be considered for HS patients with the presence of any of the following and administered immediately if a patient meets 2 or more of the following: 1) ineffective general physical cooling methods and body temperature continuously higher than 40° for over 2 h; 2) blood potassium exceeds 6.5 mmol/L; 3) CK > 5000 U/L or the rate of elevation exceeds one-fold every 12 min the presence of AKI; 4) oliguria, anuria or uncontrollable capacity overload; 5) daily increment of Scr exceeds 44.2 μmol/L; 6) uncorrectable electrolyte and acid-base imbalances. Indications for discontinuation of CRRT include: 1) stable vital signs and disease conditions; 2) CK < 1000 U/L; 3) corrected water, electrolyte and acid-base imbalances; 4) urine volume exceeding 1500 ml/d or renal function returns to normal.

## Prevention

After HS onset, it progresses rapidly, and often complicated with functional damage in multiple organs, and specific clinical treatment is limited. Unlike other critical illnesses, HS is completely preventable. The key for reducing HS-related mortality is to prevent. The occurrence of HS is closely associated with environmental, individual and training (physical activity) factors, and therefore, prevention of HS should also consider these 3 aspects. The prevention of CHS mainly emphasizes environmental and individual factors, while prevention of EHS mainly emphasizes training factors. Here, are 2 concepts related to HS prevention:

Heat adaptation: refers to the biological phenomenon that people living in a hot environment for a long time have significantly better heat tolerance than people entering the hot environment for a short duration.

Heat acclimatization: acclimatization refers to the state in which an individual adapts to a particular environment. Heat acclimatization is an acquired protective physiological response of the body to hot environment stimuli and is also known as acquired heat adaptation or physiological heat adaptation. Heat acclimatization can be produced, strengthened and lost. Once heat stimulation stops, heat tolerance will gradually weaken and return to the level prior to the acclimatization, which is called deacclimatization.

### Prevention of CHS

When the temperature exceeds 30 °C in the summer, the ratio of HS increases significantly. When the temperature does not exceed 30 °C, HS can also occur when the local temperature exceeds 30 °C because of factors such as special dress (such as poorly breathable clothes, chemical protective clothing, anti-nuclear radiation suits and fire-fighting clothing), special positions (such as high temperature operations including fire-fighting) or special environments (such as greenhouses, enclosed plants/car compartment/operation room or inside a tank). These populations should be targeted for the prevention of CHS.

CHS often occurs in the elderly, individuals with underlying disease (such as hypohidrosis and severe skin diseases), weak or bedridden individuals, infants, alcoholics or drug users, pregnant women and people who take certain drugs that affect thermoregulation (such as anticholinergics, antihistamines, antipsychotics, ß blockers and diuretics) [124]. In the summer, the living environments of these populations should be considered, e.g., air conditioners should be used to reduce the indoor temperature and clothing should be increased/decreased in a timely manner. People should drink water to avoid dehydration and seek medical advice promptly if they experience chills, diarrhea and fever. Children should not be left alone in a car or a small space.

### Prevention of EHS

#### Heat acclimatization training

Heat acclimatization training is an effective way to improve an individual’s heat tolerance [125–128]. People should undergo heat acclimatization when entering a hot area from cold or warm areas or prior to high intensity training in the early summer of each year. The environmental temperature for heat acclimatization training should start from low to high and training in extremely hot weather should be avoided. Training intensity should be increased gradually and should not exceed physiological tolerance. Training should be conducted 1-2 times each day, and each training should last 1.5-2.0 h (not less than 50 min). The total number of trainings should be no less than 6-12 times with a training period of 10-14 days.

#### Appropriate training time

Organizations and management personnel should participate in HS prevention training. Activities should be scheduled and high temperature periods should be avoided or be shortened if training in high temperature environments. The risk of HS during training can be predicted by the heat index calculated from the combination of temperature and humidity. HS occurs significantly more frequently when the heat index is > 40, and HS is extremely likely to occur when the heat index is > 55.

#### Focus on key populations

Adequate rest should be provided prior to high-intensity training. Sufficient sleep can relax the brain and all systems of the body and is an important measure for the prevention of HS. Special attention should be paid to individuals with a cold, fever, abdominal pain, diarrhea, stress, and insufficient sleep because of night duty and to new recruits, and appropriate monitoring should be provided for these people during training.

#### Timely water replenishment during training

It is extremely important to hydrate with water during training. People are extremely prone to HS with a body weight loss > 2% [6, 129]. When performing high-intensity training in a very hot and humid environment, individuals can lose 1-2 L or more of water each hour; supplementation with a sodium-containing beverage (1 L/h) is recommended [130]. Notably, drinking large amounts of normal water during exercise may cause hyponatremia, which may induce febrile convulsions and heat syncope. Isotonic saline can be taken orally when low-sodium convulsions occur, or sodium can be quickly ingested by eating pickles and drinking mineral water [131] to relieve convulsions.

#### Focus on monitoring during training

Measurement of core temperature during training for vulnerable individuals (with susceptibility factors) and those displaying abnormal presentations (pale, flushing, mental/behavioral abnormalities, physical discomfort, etc.) can prevent or detect HS early. Training should be stopped if an individual’s core temperature exceeds 39 °C and measures should be taken promptly to reduce body temperature; training can be continued when the individual’s body temperature returns to normal [131–133].

## Data Availability

Not applicable.

## Abbreviations

ACT: Activated clotting time
ALT: Alanine aminotransferase
APTT: Activated partial thromboplastin time
ARDS: Acute respiratory distress syndrome
AST: Aspartate aminotransferase
CHS: Classic heat stroke
CI: Cardiac index
CK: Creatine kinase
CK-MB: Creatine kinase isoenzyme
CR: Coagulation rate
DIC: Disseminated intravascular coagulation
EHS: Exertional heat stroke
HS: Heat stroke
LDH: Lactate dehydrogenase
PF: Platelet function
PIC: Plasmin-α_2_-antiplasmin complex.
PLT: Platelet count
PT: Prothrombin time
TAT: Thrombin-antithrombin complex
TEG: thromboelastography
TM: Thrombomodulin
TTM: Targeted temperature management
